# Measures to improve participation of registered nurses in HIV and AIDS research

**DOI:** 10.4102/hsag.v28i0.2484

**Published:** 2023-12-11

**Authors:** Justin O. Rojaye, Robert T. Netangaheni

**Affiliations:** 1 Department of Health Sciences, Faculty of Human Sciences, University of South Africa, Tshwane, South Africa

**Keywords:** AIDS, HIV, measures, nurses, participation, research

## Abstract

**Background:**

Registered nurses are crucial in lowering the complications of human immunodeficiency virus (HIV) and acquired immunodeficiency syndrome (AIDS) in Nigeria. Human immunodeficiency virus infects individuals and impairs the immune system, impairing the body’s capacity to fight bacteria, viruses, and other pathogens. Therefore, registered nurse-led initiatives are beneficial in promoting HIV and AIDS research development. However, these evidence-based treatments and professional expectations for registered nurse research creation do not represent contemporary research development on HIV and AIDS by registered nurses.

**Aim:**

This study aimed to explore measures to improve the participation of registered nurses in HIV and AIDS research in Nigeria.

**Setting:**

The study was conducted at a general hospital in Ijebu Ode, Ogun State, Nigeria.

**Methods:**

A qualitative approach design was employed. Participants of the study comprised 31 registered nurses in three focus groups.

**Results:**

This study demonstrated the importance of research in effectively reducing HIV and AIDS transmission and maintaining good practices in a hospital setting in Nigeria. However, this understanding did not translate into knowledge creation through autonomous research productivity in this context.

**Conclusion:**

The study discovered that registered nurses were primarily involved in data collection and validation, which did not result in publications. The study found that registered nurses in Nigeria are typically worried about ways to promote nurses’ engagement in research in Nigeria.

**Contribution:**

These results might be utilised to improve the supply of research services and act as a baseline for future research linked to HIV and AIDS transmission prevention in Nigeria.

## Introduction

Despite accounting for just 11% of the global population, Africa is still the epicentre of the Human Immunodeficiency Virus (HIV) pandemic. However, research on HIV and Acquired Immune Deficiency Syndrome (AIDS) by registered nurses in the continent has remained insignificant (Avert 2022). The healthcare research approach is critical to the HIV response, with registered nurses seen as significant healthcare policymakers because of their involvement in treating HIV and AIDS patients (Ogbolu et al. 2018). Therefore, it is expected that registered nurses should conduct their own research, participate in research, and use scientific evidence in their clinical practices (Avert 2022). In this sense, nurses’ substantial involvement in treating HIV and AIDS patients should continue translating into research regarding HIV and AIDS transmission in Nigeria. Studying participation by registered nurses can be a systematic way of overcoming obstacles to influential their engagement in evidence-based healthcare research-creation in HIV and AIDS transmission programmes (Rivaz et al. 2019). Furthermore, these authors argue that limiting HIV transmission would necessitate the practical application of research as the most appropriate option to address HIV and AIDS transmission in Nigeria, which would require the incorporation of multidisciplinary collaborative research involving various stakeholders, such as researchers, programme implementers, and policymakers, to accelerate research cooperation and communication among these vital stakeholders. According to Sturke et al. (2018), nurses, who play an essential role in HIV and AIDS treatment implementation, should be actively involved in healthcare research to eliminate HIV and AIDS in Nigeria.

Richter et al. (2017) agree with Sturke et al. (2018), arguing that registered nurses should be involved in innovative research efforts to battle and eventually destroy the HIV epidemic. In this respect, the International Centre for AIDS Care and Treatment Programs (ICAP 2018) attributes the high success rate in HIV programme coverage to nurses’ critical role in implementing prevention measures. As health professionals, registered nurses are essential in decreasing disease complications such as HIV and AIDS. However, even though HIV and AIDS may be effectively treated, the researcher discovered a lack of participation of registered nurses in research in the context of HIV and AIDS in Nigeria. Consequently, issues about how Nigerian registered nurses may assist in boosting nurses’ engagement in research in Nigeria arose. Registered nurses are expected to contribute to research and intellectual inquiry, but the practice-research gap remains significant despite continued efforts. Moreover, system initiatives have not addressed nurses’ concerns about their unwillingness to participate in research (Richter et al. 2017:12). Given the consequences of a lack of adequate research for registered nurses in HIV, identifying individual nurse interest in research should be a high priority for nursing leaders and academics.

Registered nurses provide a unique viewpoint to discover practice gaps, design, and assess and execute treatment to care for the patients as the only professionals with a constant presence at the bedside (Richter et al. 2027:12). Despite the proven advantages, considerable hurdles to participation in HIV and AIDS research efforts continue to exist. Nursing leaders and organisations have devised system-wide initiatives to remove obstacles that prevent registered nurses from becoming motivated. Unfortunately, system-wide methods do not address individual nursing issues (Scala et al. 2019:8). This study aims to outline a strategy for increasing nurses’ engagement in HIV and AIDS research in Nigeria.

## Study purpose

This study aimed to explore and describe the measures to improve the participation of hospital registered nurses in HIV and AIDS research in Nigeria. The following research questions guided the study:

- What are nurses’ perspectives on HIV and AIDS research in Nigeria?
- What strategies might increase nurses’ engagement in HIV and AIDS research?

## Research method and design

A qualitative technique was employed in this study. It is a technique for understanding and obtaining insight into a scenario, culture, person, or event being investigated (Silverman 2019:22). The goal of qualitative research is to look for meaning methodically. This dynamic technique combines intuitive and inductive methods to understand better what has been studied and improve interpretations (Creswell 2018:32). Researchers depend on previous experience with study venues, participants, and records (Taylor & Bogdan 2018:7). According to Hatch (2022:6), qualitative analysis is ‘the process of organising and analysing data to enable researchers to recognise patterns, identify themes, establish connections, develop explanations, provide interpretations, issue critiques, or construct ideas’. Silverman (2019:22) defines qualitative analysis as an investigation of an event by either a person or an organisation. The systematic gathering, analysis, and reporting of data create it. The qualitative approach was used for this study because it allowed for evaluating nurses’ perspectives on their involvement in HIV and AIDS research.

### Setting

The study was conducted in a general hospital at Ijebu Ode, Ogun State, Nigeria. This setting was selected because the study aimed to identify and explain strategies to promote registered nurses’ engagement in HIV and AIDS research in Nigeria by identifying and characterising the participants’ behaviours in a context where they habitually carry out their jobs (Table 1). In other words, the research was conducted in a natural context, primarily via focus group interviews, to better understand nurses’ viewpoints on research.

**TABLE 1:** Demographic profiles and characteristics of participants.

Variable	Number (N)
**Gender**	
Males	13
Females	18
**Age (years)**	
30–39	14
40–49	17
**Nursing qualification**	
Diploma	14
BScN	17
**Years of experience**	
6–10	3
11–15	14
16–20	14
**Years working at the hospital**	
1–5	14
6–10	17
**Employment status**	
Full time	31

### Sampling method

The non-probability, purposive sampling approach was utilised to recruit participants for the research. The recruitment of the participants was carried out by the researcher with assistance from the HIV and AIDS unit manager. Because this was qualitative research, the methodology used typical case, homogeneous, and criterion sampling aspects. The hospital registered nurses were chosen to participate in this study because of their health knowledge and ability to provide complete and accurate information. The inclusion criteria were full-time hospital registered nurses who have worked more than 1 year treating HIV patients and agreed to participate in the study. The target population consisted of registered nurses working full-time at the hospital. The target population was all registered nurses working in the hospital, which was over 350 nurses. The study’s sample was 31 registered nurses working in the HIV and AIDS unit. The goal was to recruit registered nurses who have experience in HIV and AIDS treatment and prevention in Nigeria and could offer rich data on nurses’ engagement in HIV and AIDS research.

### Data gathering method and process

The researcher used a semi-structured focus group interview guide with open-ended questions to gather in-depth information regarding promoting nurses’ engagement in research in Nigeria. Each focus group section lasted for 1 h. The data-gathering procedure comprises five steps forming the research’s conceptual framework. The following steps served as a framework for data gathering in the study: Stage 1 is about preparing for data, Stage 2 is about choosing data collecting criteria, Stage 3 is about assessing performance, Stage 4 is about making changes, and Stage 5 is about maintaining improvement. Data were obtained in 2022 using semi-structured interviews to allow participants to discuss their thoughts on participation in HIV and AIDS research in Nigeria. Three focus group interviews with seven participants each were held. Focus groups are appropriate when participant engagement has the potential to give broader insights that may be developed via discussion. In addition, the benefit of focus groups is that members’ perspectives may be contrasted. ‘What are nurses’ perceptions on HIV and AIDS research in Nigeria, and what strategies might be used to increase nurses’ engagement in HIV and AIDS research’ were the major questions that guided the interviews. This was followed by probing questions about the study’s goal. Data collection continued until no new information was discovered, indicating data saturation.

## Data analysis

Thematic analysis was used to identify and review themes around the measures to improve the participation of registered nurses in HIV and AIDS research development in Nigeria. According to Creswell (2018:23), data analysis begins with data management, reading and memorising, summarising, categorising, analysing, and developing a narrative that accurately reflects the case study’s story. Furthermore, Creswell (2018:23) states that in qualitative analysis, participants’ interpretations are crucial in providing the best explanations for their actions, behaviours, and thoughts. As a result, thematic analysis was chosen for this study because it is both straightforward to apply and theoretically versatile (Alhojailan 2022:8). Following the interviews, the researchers studied and transcribed the data verbatim. The researcher reviewed the transcriptions numerous times to get a broad comprehension of the data. Sections of the data that seemed to be different participant viewpoints were highlighted to construct general subjects that were shortened into themes and subthemes (Creswell 2018:24). Finally, the researcher looked for connections within the data to form a larger picture. Data analysis aids in the identification of developing patterns or themes that give a complete grasp of the topic of research (Williamson, Given & Scifleet 2018:8). The researcher used an inductive technique in which the themes included within the whole data set were found and analysed without a hypothesis.

### Trustworthiness

Trustworthiness refers to confidence in the data, interpretation, and processes used to ensure the quality of research (Polit & Beck 2021:32). The study’s trustworthiness was determined by examining its credibility, transferability, dependability, and confirmability. Building trustworthy relationships with participants throughout the recruiting process was accomplished by sticking to appointments and interview times. To build credibility, member verification was employed (Brown & Schmidt 2019:7). During member verification, the researcher returned the data to the participants to confirm the accuracy and consistency of their experiences. In addition, the researcher garnered credibility by providing a detailed overview of the processes and techniques employed in the study. This research exhibited credibility by enabling participants to offer clarifying questions. Transferability was accomplished by appropriately identifying the study’s setting and assessing the representativeness of the data. Transferability was achieved by thoroughly detailing the study strategy and approach. Authenticity was assured by establishing that the conclusions of this research exclusively represent the participants’ perspectives. Representativeness was achieved by carefully selecting registered nurses who are only working in the HIV and AIDS unit, and who gave rich, all-encompassing data that enabled saturation levels to be identified.

Consequently, only registered nurses who are working in the HIV and AIDS unit were selected to participate in the interviews. Dependability was achieved by developing a clear and complete description of the techniques, allowing the results to be checked by other reviewers. In addition, dependability was assured by using acceptable research methodologies and methodological applications. Confirmability refers to the study’s objectivity during data collection and processing (Creswell 2018:22). Confirmability was obtained by meticulously documenting the processes, methods, themes, and sub-themes. The interviewer transcribed the material verbatim and coded it to improve confirmability in this research.

### Ethical consideration

The College of Human Sciences Research Ethics Review Committee (CREC) of the University of South Africa (UNISA) approved the research (reference no.: 60825588_CREC_CHS_2022). The study followed the Code of Ethics for conducting research with human subjects. The hospital authorities and all the participants consented in writing. The participants were also guaranteed anonymity and confidentiality. The researcher obtained approval from all key parties, including the CREC at UNISA, and the hospital (research setting). Before beginning data collection, all participants provided written, voluntarily informed permission. In addition, participants were informed that they had the freedom to withdraw from the research at any time without penalty. To maintain anonymity and confidentiality, participants were given codes throughout data collection. After transcribing the data, the researcher removed it from the recorder. According to university protocol, the transcribed data will be maintained in a lock-in-a-key closet for 5 years.

## Results

The data from the focus group interviews produced two themes and seven subthemes (Table 2). The research revealed: individual initiatives and organisational initiatives. The subthemes will be examined in more detail next.

**TABLE 2:** Measures to improve participation of registered nurses in HIV and AIDS research.

Themes	Subthemes
Individual initiatives	Enhance the existing multidisciplinary team. A positive attitude by registered nurses towards research.
Organisational initiatives	Enhance communication. Provide ongoing training for nurses. Increase nurses’ incentives for participation in research. Provide adequate resources for optimal research. Incorporate research in nursing education and practice.

### Theme 1: Individual initiatives

According to all focus group participants, individual efforts should be enhanced. Individual cooperation, according to the participants, is a collaborative effort by all registered nurses to increase nurses’ engagement in HIV and AIDS healthcare research. The findings revealed two categories: improving the current interdisciplinary team and nurses’ favourable attitude towards healthcare research. Subthemes under Theme 1 are covered in detail next.

#### Subtheme 1.1: Enhance the existing multidisciplinary team

Participants stated that strengthening the multidisciplinary team will increase nurses’ engagement in healthcare research in Nigeria’s HIV and AIDS transmission context. Most participants suggested well-structured cooperation with other health professionals to increase nurses’ engagement. They recognised that all nursing units must collaborate on healthcare research. They named physicians, physiotherapists, pharmacists, and psychologists as possible members of the multidisciplinary collaborative team. The following statements support the discovery:

‘Holding a weekly interdisciplinary research conference might increase involvement in research by sharing fresh ideas, pushing others to participate, and resolving any problems.’ (Participant 1, female, 43 years)

‘By participating in the research committee, registered nurses may strengthen their abilities to collaborate as a team in research.’ (Participant 2, female, 33 years)

‘Every nursing unit must form a research committee, and registered nurses must make every effort to attend committee meetings as required.’ (Participant 6, female, 39 years)

According to most of the participants’, several disciplines in healthcare may operate more effectively as a team to assist in enhancing healthcare research by establishing interdisciplinary cooperation, accepting working together, and respecting one another’s opinions (World Health Organization [WHO] 2015:16). For example, nursing care for HIV and AIDS patients necessitates collaboration with the primary care provider (physician) and other disciplines engaged in continuous care (Anon 2021:286). Furthermore, the interdisciplinary team fosters a favourable atmosphere and strong interactions among registered nurses (Comer & Rao 2019:66).

#### Subtheme 1.2: A positive attitude by registered nurses towards research

Although most of the participants in this survey reported interest in HIV and AIDS research, but they had never participated in it. Even those who had previously participated were compelled to participate again. Participants indicated few impediments to HIV research and perception of healthcare research. The following statements support the discovery:

‘Registered nurses must have a favourable attitude toward research since we deal with them daily.’ (Participant 7, female, 46 years)

‘If registered nurses have a positive attitude toward research, more nursing will be able to be shared among nurses, and new registered nurses will benefit from the experience of older nurses.’ (Participant 5, female, 43 years)

‘Without a positive approach in research, the nursing profession would be overlooked worldwide.’ (Participant 9, female, 40 years)

The data emerged that the capacity of nursing practitioners to refresh their knowledge and use up-to-date, evidence-based treatments in performing their tasks is critical to providing excellent nursing care to patients. Participants stated that fresh evidence is discovered daily in healthcare (Huiting 2017:4). Participants’ statements were also supported by Huiting (2017:4); nurses’ ability to adapt to these changes will substantially influence the quality and safety of the healthcare provided. According to Huiting (2017:4), new knowledge must be assimilated and utilised in healthcare delivery following the basic standards of nursing. In addition, participation in healthcare research allows for evidence in care delivery.

### Theme 2: Organisational initiatives

According to Jamison and Edwards (2019:51), healthcare research initiatives are relevant interventions that promote nurses’ engagement in healthcare research. According to Registered Nurses Association of Ontario (RNAO 2018:44), organisations must understand that all registered nurses have the right to the most acceptable HIV and AIDS research in their workplace. The organisational initiatives revealed five categories, and further information on each is discussed as follows:

#### Subtheme 2.1: Enhance communication

Participants indicated improved communication as essential in increasing nurses’ engagement in HIV and AIDS research. They suggested effective communication techniques, such as proper communication among nurses, enabling them to exchange ideas, and urge others to engage in HIV and AIDS research. The following statements corroborate the findings:

‘Keeping open and effective communication amongst registered nurses throughout the creation of research and policy may aid in boosting nurses’ engagement in research.’ (Participant 10, male, 41 years)

‘I need to interact with other registered nurses during research in a manner that others can relate to by asking basic and straightforward research questions.’ (Participant 8, female, 47 years)

The participants’ statements that open communication in healthcare research teams leads to better results were supported by several studies (Kaasalainen et al. 2019:667). According to Kourkouta and Papathanasiou (2018:65), communication skills are critical and essential in nursing. Nurses’ jobs require talking with patients from various educational, cultural, and social backgrounds. Therefore, registered nurses must be practical, compassionate, and professional when communicating with colleagues and patients (Kourkouta & Papathanasiou 2018:66).

#### Subtheme 2.2: Provide ongoing training for nurses

Participants recommended providing staff with ongoing training opportunities and support in HIV and AIDS research in all the focus groups. They believed this could contribute to nurses’ participation in HIV research and future growth. In addition, all participants emphasised the need to understand and be aware of existing HIV and AIDS research protocols. The statements that follow confirm the finding:

‘You can only know what you are taught and trained for; our management must organise in-service research training to assist us to enhance our abilities.’ (Participant 14, female, 53 years)

‘For me to participate, I need to be taught the new methodologies and kept up to speed on the newest research changes.’ (Participant 16, female, 38 years)

According to many participants, several studies have demonstrated the need for continued HIV and AIDS research for registered nurses (Kaasalainen et al. 2019:667). In addition, data from the study showed that in-service training that empowers registered nurses might increase their engagement in HIV and AIDS research while also improving patients’ quality of life (Chaghari et al. 2017:32). New employees must be orientated to the organisation’s research, processes and practices, and continuous professional in-service training to support the adjustment and positive early experiences of new hires (RNAO 2018:44).

#### Subtheme 2.3: Increase nurses’ incentives for participation in research

Participants recommended that increasing nurses’ engagement in HIV and AIDS research projects via incentives might increase their participation. They argued that registered nurses should be included in decision-making regarding research, including the possible prevention of HIV and AIDS transmission in Nigeria. Participants acknowledged nurses’ contributions to critical healthcare research initiatives, particularly for HIV and AIDS patients in Nigeria. The verbatim quotations that support the conclusions are as follows:

‘Registered nurses are an excellent source of healthcare knowledge throughout HIV and AIDS therapy; I simply believe we need to get them more engaged in research by giving them the incentive to participate in research.’ (Participant 18, female, 43 years)

‘I will advocate for registered nurses to be given incentives and funds so that they may recognise the value of research and participate.’ (Participant 15, female, 36 years)

‘As you may know, doctors get a lot of incentive for all the little things they do outside of their typical duties; I’m curious why this isn’t the case for nurses.’ (Participant 11, female, 43 years)

Participants identified several other potential enablers to reduce barriers and improve their ability to participate in HIV and AIDS research, including compensated time for research, academic collaborators, research support staff, mentorship, and electronic health records, similar to other research (Bakken et al. 2019:3). Data showed that creating a realistic budget that covers the expense of the study is one of the essential techniques for registered nurses working in HIV and AIDS research (Bakken et al. 2019:3).

#### Subtheme 2.4: Provide adequate resources for optimal research

Participants stated that registered nurses need appropriate resources such as computers, time and relevant support for HIV research and the ability to employ them. Participants also recommended that increasing adequate research resources, such as Internet services and time outside working hours, would improve nurses’ research quality in Nigeria. The following statements support the discovery:

‘In research, I need to be given resources to participate in research for me to be interested in research.’ (Participant 12, female, 43 years)

‘Adequate research resources are required for nurses’ engagement in research; thus, our management must create a provision for resources to enable optimum research, which will lessen the obstacles I confront.’ (Participant 13, female, 53 years)

‘Many of our registered nurses are from other cultures; developing a cultural resource for research is critical to improving their engagement in research.’ (Participant 17, male, 43 years)

According to Olajumoke, Yemisi and Gabriel (2021:3), the availability of educational resources has always been regarded as an essential and integral part of nursing administration, geared towards the improvement of all other factors in the HIV research and nursing process, thereby ensuring quality service delivery by registered nurses to society. As a result, the success of nursing in Nigeria is dependent, among other things, on effective nursing administration with good leadership, proper time management in the healthcare system, adequate financial resources allocated to healthcare, regular training and re-training of nurses, perfect interrelationship with the community, and creative use of the available resources in the healthcare system (Olajumoke et al. 2021:3).

#### Subtheme 2.5: Incorporate research in nursing education and practice

Participants felt integrating research into nursing education, and practice may boost nurses’ morale, resulting in more research engagement. They emphasised the organisation’s need to use effective research methodologies to increase the quality of research. The following exact quotes support this conclusion:

‘If our management implements research into practice, nurses’ morale and feeling of belonging will improve. Therefore, nurses’ engagement in research yields the most outstanding results.’ (Participant 19, female, 40 years)

‘We must include standardised research techniques into nursing education and practice to lessen HIV and AIDS complications.’ (Participant 20, female, 43 years)

Kaasalainen et al. (2019:668) confirmed the data from the study that established the advantages of integrating research into nursing education and practices. However, RNAO (2018:34) states that registered nurses must have an acceptable workload to offer sustained research. Furthermore, employers are accountable for providing suitable opportunities for registered nurses to participate in research.

## Discussion

Methods for allowing registered nurses to challenge present procedures must also be considered. To offer high-quality patient care and results, registered nurses at all levels of healthcare must work together. Therefore, research efforts may aid in the formation of productive collaborations. Mentoring programmes that encourage using nurses’ ideas as research subjects for literature reviews, evidence synthesis, and implementation are effective. However, care should be taken to avoid the impression that clinical priorities and physician research limit nursing research (Hagan 2018:8). The collaboration will be encouraged via multidisciplinary education, supervised participation, journal clubs, and research activities, which will help to foster a research friendly culture and increase communication (Cline et al. 2017:12). The impact of registered nurses on health research protects care quality by giving access to essential resources and opportunities (Arabi et al. 2017:8). This is a novel and vital idea for nursing; nevertheless, research studies on nurses’ research impact in the healthcare sector lack a fundamental conceptual grasp of what this notion signifies (Dowswell, Dowswell & Young 2017:22). In addition, registered nurses have varied viewpoints on healthcare issues and influence healthcare legislation differently. However, registered nurses will recognise the significance of research in the health sector and their effect on this process and patients’ outcomes if they understand nurses’ research to influence nursing practice on HIV and AIDS treatment concepts (Dowswell et al. 2017:23).

The findings give an insight into nurses’ obstacles, perceptions, knowledge, and engagement in healthcare research, adding to the body of knowledge on nurses’ perspectives on healthcare research (Huiting 2017:4). Despite the hurdles, the desire for healthcare research remains one of the top concerns for registered nurses in many contexts, requiring registered nurses to give practical, safe, and efficient care (Huiting 2017:4). Healthcare systems are evolving and changing at a fast pace. Registered nurses should implement these adjustments as part of this system (Arabi et al. 2017:8). Instead of implementing health research, registered nurses must influence them to reach this aim. Then, to better govern their practice, they must take an active role in formulating health research. Nurse leaders have an essential part (Dowswell et al. 2017:23). To solve professional issues, they must learn research-making skills. Nurse executives bring unique and critical viewpoints on health issues because of their ideals, professional ethics, advocacy abilities, and experiences (Arabi et al. 2017:9). In recent years, nurses’ presence, status, and impact in the workplace have expanded. Registered nurses must identify issues proactively and engage with other decision-makers to improve healthcare research (Dowswell et al. 2017:22). They should understand the various power levels in their organisations and who controls the healthcare resources. Consequently, we can assert that registered nurses must actively participate in policies affecting patients, families, themselves, and the healthcare system (Dowswell et al. 2017:23).

According to Hagan (2018:8), it emerged from the study data that positive (e.g., monetary or academic incentives) and negative (e.g., worries about clinical productivity) feedback gained from clinical research involvement reinforce variables that might encourage nurses’ engagement in research. Professional advancement, continuous medical education, engagement with university researchers, and acknowledgement as a reward for members or research partners were all-important motivations. A well-established programme involving academic and financial incentives enables registered nurses to construct an active research programme, which is presently unavailable to most nurses. As a result, adopting an innovative way to give protected time and secure funds to boost clinical nursing research, such as grants and industrial financing, may provide temporary assistance (Stutzman et al. 2016:9). The impact of registered nurses on health research preserves patient safety, improves care quality, simplifies access to needed resources, and promotes excellent healthcare. As a result, the research’s impact on nursing is novel and necessary. Still, there is a lack of conceptual clarity about what research impact on nursing entails, Dowswell et al. (2017:24); for example, this study found that most primary care groups at primary care centres spoke with local registered nurses about the needed fields of care services and considered that contact with registered nurses was successful. However, compared with other health professionals, the findings of this study regarding nurses’ participation in HIV and AIDS research could influence different health professions in preventing HIV and AIDS transmission in Nigeria (Dowswell et al. 2017:23).

Establishing expectations early in a nursing career fosters a culture of clinical research (Schuessler et al. 2018:8). Nursing education programmes that require merely a study of the literature on any issue fall short of advancing registered nurses beyond academic expertise. When education programmes emphasise developing abilities that not only foster research but also give implementation solutions, confidence, self-fulfilment, and happiness are promoted (Scala et al. 2019:6). According to the findings by Aksoy et al. (2018:4), most registered nurses are aware of volunteering to participate in research and safety issues, but many are unfamiliar with rules or study design. Participating in current initiatives and supporting local change might be a terrific way to start. Registered nurses need chances to participate and foster self-fulfilment to maintain engagement and enhance practice (Scala et al. 2019:10). Finally, including research involvement in yearly performance assessments may encourage future research initiatives (Scala et al. 2019:10).

## Conclusion

Registered nurses should contribute to clinical research and support the delivery of high-quality care. Simultaneous clinical practice and research increase the quality of care provided to HIV and AIDS patients locally, nationally, and worldwide. While many researchers have attempted to overcome constraints to shrink the gap between research creation and practice (e.g., via the implementation of education-based treatments), efforts to bridge the ‘gap’ have been only partly effective at best. Such a schism leads to the provision of treatment that is either unnecessary, ineffective, inefficient, or conflicting with other procedures; the inference being that patients get subpar care. Given the potential consequences of the HIV and AIDS research-practice gap for quality and safety, identifying measures to close the gap should be a priority for nurse leaders and academics. Nursing research goals, persuading registered nurses to engage in HIV and AIDS research, a good culture, and leadership support and resources to carry out HIV and AIDS research activities are all potential answers. In addition, it is critical to strengthen nurses’ capacity building to engage in HIV and AIDS research development. Certain areas of improvement (e.g., improved communication, training, incentives, interdisciplinary team approach, and a positive attitude towards research development) are recommended to boost nurses’ research development practice.

### Limitation

This study looked at ways to increase nurses’ engagement in research in Nigeria. However, precautions were taken to mitigate the possible impact of the restriction on the study’s richness. Furthermore, the study results cannot be generalised to broader groups or populations because of the small sample size. For example, if the researcher had employed a large sample that covered a large geographical area, the researcher would have acquired a variety of viewpoints and estimations on the study subject.
